# Olanzapine for the Prevention and Treatment of Chemotherapy-Induced Nausea and Vomiting: A Review to Identify the Best Way to Administer the Drug

**DOI:** 10.3390/curroncol29110650

**Published:** 2022-10-31

**Authors:** Xiao-Ling Zhang, Jie-Er Ying

**Affiliations:** 1 Wenzhou Medical University, Wenzhou 325000, China; 2 The Cancer Hospital of the University of Chinese Academy of Sciences, Zhejiang Cancer Hospital, Hangzhou 310022, China

**Keywords:** olanzapine, CINV, mechanism, dosage, time, routes

## Abstract

Common treatment methods for malignant tumors include surgery, chemotherapy, radiotherapy, immunotherapy, targeted therapy, etc., among which chemotherapy plays an important role. However, chemotherapy brings corresponding side effects while killing tumor cells, and nausea and vomiting are the most common adverse reactions induced by chemotherapy. It not only affects the patient’s appetite, resulting in malnutrition and electrolyte disturbances, but also reduces the patient’s compliance with treatment, which further aggravates the disease. Thus, it is important to quickly prevent and cure nausea and vomiting induced by chemotherapy (CINV). In addition, with the continuous development of medicine, more and more antiemetic drugs have been developed. At present, the most common antiemetic agents for chemotherapy-induced nausea and vomiting are NK-1R antagonists, 5-HT3R antagonists, and dexamethasone. Surprisingly, olanzapine, often used as a psychotropic drug, has been found to be an effective antiemetic and is similar to other regimens on the safety of medicine. However, although there are numerous studies on the antiemetic effects of olanzapine, its comprehensive application remains unclear. Therefore, this review will elaborate the antiemetic effect of olanzapine in terms of the antiemetic mechanism and the safety, economic cost, dose, administration time, and drug delivery aspects.

## 1. Introduction

Chemotherapy is one of the most important methods in the treatment of cancer at present, and is known as one of the three major treatment methods for cancer, along with surgery and radiotherapy. It is a systemic therapy which can effectively control a tumor’s growth or kill it, but chemotherapy will damage normal cells while affecting tumor cells, bringing a variety of adverse reactions and side effects. Additionally, CINV is a common untoward reaction to cancer therapy [1]. The incidence rate of CINV has been estimated to be as high as 70–80% without appropriate antiemetic drugs [2]. Patients’ quality of life can be deteriorated by CINV, which may lead to poor compliance to chemotherapy, and seriously affect the therapeutic efficacy. In the course of antitumor therapy, antitumor drugs can generally be divided into high, moderate, low, and mild emetic risk grades. Among patients who do not receive prophylactic antiemetic agents, the guidelines point out the incidence of vomiting induced by highly emetogenic chemotherapy (HEC) is more than 90% within 24 h after chemotherapy; for moderately emetogenic chemotherapy (MEC), this rate is 30–90%, for low-emetogenic chemotherapy (LEC), this rate is 10–30%, and for mildly emetogenic chemotherapy, this rate is less than 10% [3]. Before olanzapine is used for antiemetic purpose, the most commonly used antiemetics for CINV are 5-HT3R antagonists, NK-1R antagonists, and dexamethasone [4]. For HEC, the combination of 5-HT3R antagonists, neurokinin-1R antagonists, and dexamethasone is recommended. For MEC, the combination of 5-HT3R antagonists and neurokinin-1R antagonists is suggested. Additionally, for LEC, a single antiemetic agent such as a 5HT3 receptor antagonist, dexamethasone, or a dopamine receptor antagonist may be considered. Antiemetic drugs should not be routinely taken by patients receiving chemotherapy with a minimal emetic regimen [3,5]. Despite the existence of many medicines for the control CINV and extensive research studying it, patients still experience nausea and vomiting; the management of CINV remains a significant challenge [6].

Olanzapine is a second-generation psychotropic for schizophrenia and bipolar disorder which belongs to the benzodiazepine class of psychotropics [7,8]. It was first found to be effective in preventing and treating nausea in palliative care settings and among patients with opioid-induced nausea according to a review published in 2003 [9]. Additionally, it was reported to have antiemetic effects in 2005 [7]. Since the early 2000s, olanzapine has been suggested to be effective for antiemetic purposes, and the Multinational Association for Supportive Care in Cancer (MASCC) and the National Comprehensive Cancer Network (NCCN) recommended that olanzapine could be used for the prevention and treatment of CINV [10]. Olanzapine can act on a variety of receptors, including those for adrenalin, dopamine, serotonin, histamine, and muscarine. Additionally, such a mechanism of action is deemed likely to control nausea and vomiting associated with chemotherapy [11]. The most common side effect of olanzapine is drowsiness, which is well-tolerated by patients; moreover, few other serious adverse reactions are reported [10]. A systematic review and meta-analysis of early clinical experiments including seven studies conducted by Ronald showed that 84 patients receiving HEC, 58 receiving MEC, and 44 receiving HEC or MEC were enrolled, and the total complete response (CR) upon using olanzapine in the acute, delayed, and overall periods was 97.2% (138/142), 83.1% (118/142), and 82.8% (130/157), respectively [12]. In addition, a phase II study executed by Kazuhisa et al. aimed to explore the effect of olanzapine on the prevention of cisplatin-based CINV among patients with thoracic malignancy, the results showed that CR rates for acute, delayed, and whole phases were 100% (95% CI: 0.89–1.00), 83% (95% CI: 0.66–0.93), and 83% (95% CI: 0.66–0.93), respectively, after the addition of olanzapine [13]. Additionally, in a review which included 464 participants from five studies about CINV shows that olanzapine may increase somnolence and fatigue (RR: 2.33, 95% CI: 1.30–4.18) [14].

**Figure 1:**
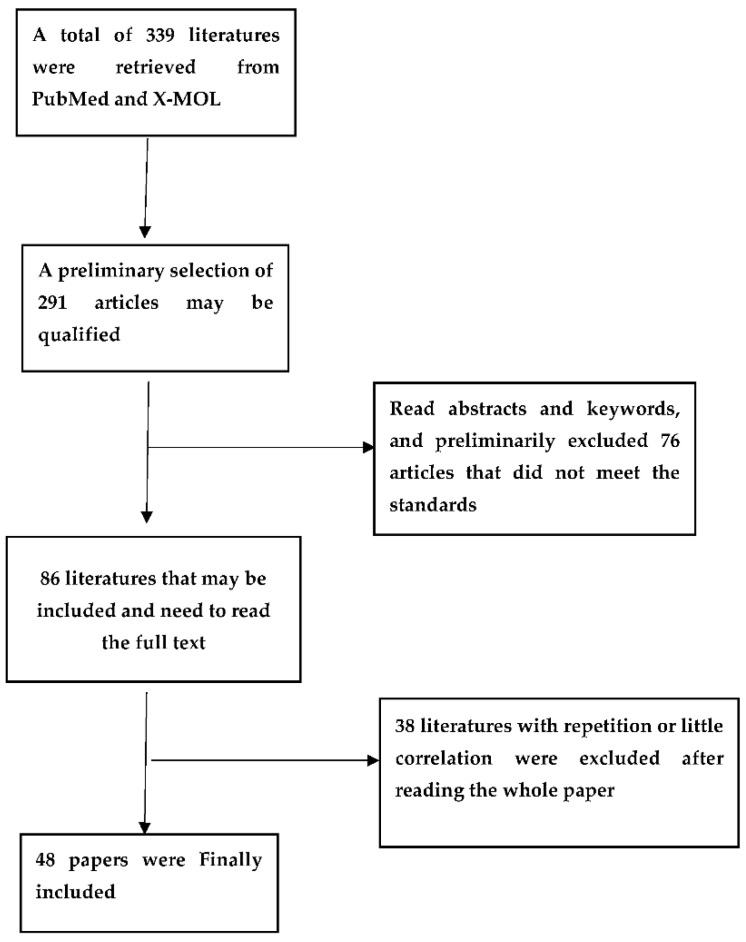
Flow diagram for the literature selection.

According to information, extensive studies have focused on the role of olanzapine in the prevention and rescue of CINV, and olanzapine is increasingly used for antiemetic purposes in reality, but is currently mostly used by patients with psychiatric and acute diseases. Additionally, some findings about the best method of administration are not fully understood. In order to fully understand the use of olanzapine, this review analyzed and summarized findings from the literature (Figure 1) to explore meaningful and comprehensive drug-delivery methods of olanzapine, especially in terms of its antiemetic mechanism, dosage, drug administration time, and routes, which are more beneficial in reducing the nausea and vomiting which are related to chemotherapy.

## 2. Pathogenesis of CINV and the Antiemetic Mechanism of Olanzapine

Chemotherapy-induced nausea and vomiting are common adverse reactions among patients with malignant tumors [15]. The mechanism of CINV involves several neurotransmitters and receptors in the central nervous system and the gastrointestinal tract, such as 5-hydroxytrypotamine and its receptors, dopamine and its receptors, substance P, and the neurokinin-1(NK-1) receptors. [16]. Chemotherapy drugs can affect intestinal chromaffin cells through blood circulation or influence intestinal mucosa directly through the enteric cavity to release serotonin, and serotonin can activate the chemoreceptor trigger zone to stimulate the release of more neurotransmitters. These neurotransmitters provoke the vomiting center, inducing movement in the esophagus, diaphragmatic muscle, and abdominal muscle, and increasing the secretion of the salivary glands at the same time; these provocations lead to the occurrence of vomiting. Meanwhile, substance P binds to the NK-1 receptor to contract smooth muscle, which can also cause vomiting. In addition, histamine, acetylcholine, and other neurotransmitters are involved in vomiting as well [17].

Olanzapine could be used as an antiemetic, and it was first found to be efficient in the prevention and treatment of nausea for patients with palliative care according to six cases, each patient in the cases exhibited marked improvement when treated with olanzapine [9,18,19]. Because of its mechanism of blocking multiple neurotransmitter receptors, olanzapine can be used as a single broad-spectrum antiemetic for nausea and vomiting caused by multiple or non-specific factors among patients with advanced cancer or for comfort care at the end of life [20]. Since the early 2000s, the antiemetic efficacy of olanzapine has been recommended [10]. Additionally, it can inhibit multiple neurotransmitter receptors, such as serotonergic 5-hydroxytryptamine (5-HT) type 2a, type 2c (5-HT2c), type 3 (5-HT3), and type 6 (5-HT6) receptors; dopaminergic D1, D2, D3, and D4 receptors; catecholamine alpha1 adrenergic receptors, histamine H1 receptors, and acetylcholine muscarinic receptors [21]. These receptors, related to specific neurotransmitters as mentioned above, can be blocked by olanzapine to exert antiemetic efficiency, particularly 5-HT2c and 5-HT3 and dopaminergic D2 [10,22]. A clinical study was conducted by Kazuki et al. to evaluate the prophylactic effect of an antiemetic regimen containing olanzapine on carboplatin-induced nausea and vomiting; a total of 33 patients with newly diagnosed lung cancer were enrolled; the overall CR was 93.3% (95% CI: 80.4–98.3%), CR was 100% (95% CI: 89.6–100%) in the acute phase, and CR was 93.9% (95% CI: 80.4–98.3%) in the delayed phase. Furthermore, questionnaires were used to assess patients’ nausea in this study; the results showed that the incidence of nausea was 3.0% in the acute phase, 15.2% in the delayed phase, and 18.2% in the total phase. The nausea rate was significantly reduced after using olanzapine, indicating that olanzapine has an excellent effect on the prevention of CINV, which cannot be achieved without olanzapine [21].

## 3. Safety of Olanzapine in the Prevention and Treatment of CINV

Olanzapine has good safety and low incidence of adverse reactions in the prevention and treatment of CINV. The most-reported side effects are drowsiness and dizziness when olanzapine is used to treat nausea and vomiting caused by chemotherapeutic drugs; however, these untoward reactions are not obvious, so there is no need to discontinue olanzapine’s use, and remarkable extrapyramidal reactions have not been reported yet [20]. In a trial performed by Kazuhisa et al., evaluating the efficacy of olanzapine in combination with standard antiemetic therapy for the prevention of HEC, a total of 30 patients with malignant thoracic disease were enrolled. Additionally, the experimental results showed that there were no grade 3 or 4 adverse events during treatment. For symptoms, there were 20 patients (67%) with grade 1 constipation, 16 patients (53%) with grade 1 hiccups, 4 patients (13%) with grade 1 sleepiness, and 1 patient (3%) with grade 2 hiccups. For biochemical test results, elevated grade 1 alanine aminotransferase levels were observed in 11 patients (37%); there was no incidence of hyperglycemia or increased creatine phosphokinase [13]. Breakthrough CINV is defined as vomiting and/or nausea caused by chemotherapy after the use of guideline-directed prophylactic antiemetics [23]. Among patients undergoing HEC, 30–40% may develop breakthrough CINV, despite the use of guideline-directed prophylactic antiemetic agents [24]. In a phase III study, 108 evaluable patients with breakthrough CINV were randomized to oral olanzapine or metoclopramide, and monitored the occurrence of nausea and vomiting within 72 h. The results showed that 39 of 56 (70%) patients treated with olanzapine had no vomiting and 38 (68%) had no nausea, compared with 16 of 52 (31%) patients; 12 (23%) patients had no nausea when treated with metoclopramide (*p* < 0.01). This suggests that olanzapine is more effective than metoclopramide in controlling breakthrough CINV among patients with HEC, and it may become an effective antiemetic drug for breakthrough CINV [25]. In addition, no grade 3 or 4 toxicity was observed in phase III trials of olanzapine at 5–10 mg, before and 3–4 days after chemotherapy [26,27,28,29]. Specifically, there was no significant dizziness, drowsiness, extrapyramidal reactions, or cathisophobia. Short-term (up to 5 days) use of small doses (5–10 mg) of olanzapine to prevent CINV is unlikely to produce endocrine or metabolic side effects [20]. Additionally, olanzapine has a lower cost. The expected cost of the olanzapine regimen is USD 325.24 versus USD 551.23 for the non-olanzapine regimen. Meanwhile, the expected utility/index is 0.89 for the olanzapine regimen versus 0.87 for the non-olanzapine regimen [30]. This can reduce the financial burden on the families of cancer patients.

## 4. Antiemetic Effects of Olanzapine in Different Doses

When olanzapine is used to combat tumor-induced nausea and vomiting, the most-reported adverse reaction is drowsiness [20]. A large number of studies have analyzed the dosing efficacy of olanzapine in the application of antiemetic, particularly the comparison between 10 mg and 5 mg. In a meta-analysis conducted by Dong-Yang Wang et al., 12 studies from the database published before 18 April 2021 were eventually included; study subjects were adult cancer patients receiving HEC or MEC. This review aimed to analyze the effects of 10 mg olanzapine and 5 mg olanzapine on the prevention and treatment of CINV, and only valid data were listed; they showed the therapeutic effect between different doses and different grades of emetogenic regimen (Table 1). The results suggested that 10 mg olanzapine might be the first choice for patients with HEC, and 5 mg might be the first choice for patients with MEC or without significant CINV risk. Both 10 mg and 5 mg of olanzapine had good prophylaxis in the delayed and overall phase, which is possibly due to the lower incidence of vomiting and nausea in the acute phase. However, olanzapine 5 mg may be a better choice for the prevention of CINV because of its low “lethargy” response [31]. In addition, Hironobu et al. performed a large phase III trial; in this research, 710 cisplatin-treated patients with malignancies (excluding those with hematopoietic malignancies) from 26 hospitals in Japan were recruited. Patients were randomly assigned to 5 mg olanzapine or placebo; all of them received triple antiemetic therapy with 5-HT3 receptor antagonist, NK-1 receptor antagonist, and dexamethasone. The study aimed to assess the antiemetic effect when 5 mg olanzapine was added to the triple therapy. The results showed that the CR and CC in olanzapine group were both higher than those in the placebo group (Table 2). It was shown that patients receiving olanzapine 5 mg plus triple therapy had significantly improved antiemetic efficacy, and olanzapine 5 mg in combination with 5-HT3 receptor antagonist, NK-1 receptor antagonist, and dexamethasone may become a new standard antiemetic treatment option for patients treated with HEC [32]. Similarly, 153 patients who received cisplatin were enrolled in a phase II clinical study conducted by Takako; the results showed that the CR for delayed period was 78% (80% CI: 70.3–83.8, *p* = 0.01) in the 10 mg olanzapine group and 86% (80% CI: 79.2–90.7, *p* < 0.001) in the 5 mg olanzapine group; incidence of somnolence was 53.3% and 45.5%, respectively [33]. This also suggests that both 10 mg and 5 mg of olanzapine significantly improved delayed vomiting, dramatically reduced the frequency and duration of nausea in high-risk patients, and 5 mg olanzapine causes less somnolence [24,25,26,27,28,29,30,31,32,33,34,35,36].

Moreover, in regard to the use of 5 mg and 10 mg olanzapine, if patients experience symptoms of nausea and vomiting after 5 mg olanzapine, a dosage of 10 mg should be considered to implement [37]. In addition, the dosage of olanzapine varies when it is used for different purposes. When used in the prevention of CINV, the dosage of olanzapine is usually 10 mg orally/daily, and it starts on the day of chemotherapy and lasts from day 2 to day 4. If the treatment starts the day before chemotherapy, olanzapine 5 mg is suggested and lasts from day 1 to day 5 [20]. Additionally, 10 mg olanzapine also as a rescue antiemetic for breakthrough CINV [38]. In advanced cancer, the initial dose is lower in the treatment of tumor-induced nausea and vomiting, and 1.5–2.5 mg is usually recommended [20]. Olanzapine is administered orally at 5 mg/day for cancer-related anorexia. Higher doses are usually lower than those used for mental health, with a maximum daily dose of 20–60 mg.

The circadian rhythm of the body will change the pharmacodynamics and pharmacokinetics of drugs in vivo, making the blood concentration, bioavailability, metabolism, and excretion of drugs change in a circadian rhythm. In addition, the purposes and requirements of medication use and the functional status of the individual’s gastrointestinal tract can also affect the time of drug administration. Olanzapine has an approximate 85% oral bioavailability, which is independent of food intake, and its maximum plasma concentration is reached 5 h after oral administration [39,40]. If olanzapine is injected intramuscularly, it can reach peak blood concentrations in less than 45 min as a result of being absorbed rapidly [10]. The half-life (T1/2) of olanzapine is 21–54 h, and the clearance rate (Cl) is 12–47 L/h [39]. Simultaneously, drowsiness is a major adverse reaction in the treatment of nausea and vomiting with olanzapine [25]. Olanzapine can increase the total sleep time, sleep period time, and sleep efficiency, and can reduce sleep onset latency. Additionally, it also leads to more physiological changes, especially an increase in slow-wave sleep and a more intense increase in rapid eye movement sleep. A study carried out by Michael et al. to explore the effects of olanzapine on sleep among patients with schizophrenia showed that the efficiency of sleepiness had increased from 83% at baseline to 95% at 2 weeks and to 97% at six weeks after using olanzapine [41]. Moreover, the time to reach maximal blood concentration of olanzapine is 3–5 h, as mentioned in a phase III study; therefore, when olanzapine is administered after dinner, the blood concentration of olanzapine can reach a peak while the patients fall asleep, which is beneficial for sleeping [32]. Thus, it may be a good choice to take a dose of olanzapine 5 h before bedtime. However, previous clinical trials have not described the optimal time to administer olanzapine in detail. Surprisingly, it should be noted that “drowsiness”, which is a side effect of olanzapine, might mitigate the “insomnia” effect of dexamethasone, reducing the incidence rate of insomnia by 10.7% [42]. The evidence for administering olanzapine at bedtime is insufficient; more research could be done on this in the future.

## 5. Effects of Olanzapine on Different Administration Routes

Different dosage forms of the same drug can make its action speed, intensity, duration, side effects, and toxic intensity different. Olanzapine can be administered in different forms [43]. Commercially, olanzapine includes oral and intramuscular preparations [40]. Currently, olanzapine is approved by the FDA for intramuscular and oral use only, and intramuscular preparations have two forms: one in immediate release form and the other in long-acting form. The immediate release form for intramuscular administration is approved by the FDA only for use in acute agitation associated with schizophrenia and bipolar I mania; the long-acting form is approved for use in the maintenance therapy of schizophrenia [44,45]. Olanzapine is absorbed well orally and has a bioavailability of about 85% [40]. In the treatment of agitation associated with schizophrenia, switching from intramuscular injections to oral preparations may help treat the disease in the long term and improve patients’ compliance to treatment [46]. Previous studies have shown that almost all patients who received intramuscular injections of olanzapine experienced agitation [47]. A study exploring the efficacy and safety of oral versus intramuscular olanzapine in schizophrenia indicated that intramuscular injection of 10 mg olanzapine effectively reduced acute agitation within 24 h among patients with schizophrenia. When switching from intramuscular injection to oral administration, the reduction in irritability lasted up to four days after oral administration, and patients who took olanzapine orally did not have dystonia, which could be induced by intramuscular injection [46]. As for the intravenous injection, the literature almost exclusively mentions the use of intravenous olanzapine among patients with anxiety in the emergency department [48]. A trial to study the efficacy of intramuscular and intravenous olanzapine administration indicated that, among 295 patients receiving intravenous olanzapine, there were 11 respiratory depression patients and 2 patients needed intubation. Among 489 patients receiving intramuscular olanzapine, there were 10 respiratory depression patients and 5 patients needed intubation. There were 6 patients in the intravenous group and 2 patients in the intramuscular group who experienced nonrespiratory complications, [44]. These data suggest that the risks associated with intravenous and intramuscular olanzapine are similar. Additionally, intravenous olanzapine could be a treatment worth considering, as it may reduce the potential risk of drug-induced arrhythmias [48]. Among patients with acute disease, it is safe to administer olanzapine intravenously, and it also may shorten emergency room time when compared with intramuscular injection, but further research is needed to confirm this [44]. At present, olanzapine is mainly used by patients with psychotic symptoms, but it has also been gradually applied for CINV, and intravenous injection is not yet approved, so more studies could be conducted in the future.

## 6. Conclusions

In summary, it is clear that the prevention and treatment of CINV are vitally important. Not only does this lead to a better curing effect, but also improves people’s quality of life. Previous studies have shown that olanzapine has a satisfactory effect on CINV, with a low economic cost and few adverse reactions; these findings are good news for the medical circle and cancer patients. Nevertheless, the use of olanzapine for antiemetic purposes is still not fully understood. In this review, a large number of studies were collected to comprehensively evaluate the optimal administration of olanzapine from aspects of antiemetic mechanism, safety, economic cost, different dose effects, optimal administration time, and different delivery routes. The review found that olanzapine doses and routes of administration may vary depending on the methods and purposes of its use, and that giving olanzapine before bedtime may be a good choice. However, evidence for olanzapine administration before bedtime remains weak and intravenous olanzapine has not yet been approved. The pathways of administering olanzapine studied in this review are mainly from psychiatric and acute patients, and data on antiemetic effects are still lacking. Therefore, further research could be carried out on these respects, and this review is expected to provide insights for the management of CINV.

## Figures and Tables

**Table 1:** CR and CC with different doses of olanzapine for different emetogenic grades.

Chemotherapy Regimens	The Dosage of Olanzapine	CR			CC		
		Acute Period	Delayed Period	Overall Period	Acute Period	Delayed Period	Overall Period
HEC	10 mg	RR = 1.29, (95% CI = 1.07, 1.56); RD = 0.18	RR = 1.64, (95% CI = 1.31, 2.06); RD = 0.29	RR = 1.96, (95% CI = 1.54, 2.49); RD = 0.34	RR = 1.40, (95% CI = 1.07, 1.82); RD = 0.16	RR = 1.92, (95% CI = 1.20, 3.08); RD = 0.19	RR = 2.24, (95% CI = 1.31, 3.81); RD = 0.22
MEC	10 mg	/	RR = 1.34, (95% CI = 1.15, 1.56); RD = 0.21	RR = 1.33, (95% CI = 1.14, 1.56); RD = 0.2	/	RR = 1.35, (95% CI = 1.09, 1.67);/	RR = 1.37, (95% CI = 1.09, 1.70);/
	5 mg	RR = 1.07, (95% CI = 1.02, 1.12);/	RR = 1.20, (95% CI = 1.10, 1.32); RD = 0.13	RR = 1.23, (95% CI = 1.11, 1.35); RD = 0.14	RR = 1.07, (95% CI = 1.02, 1.12);/	RR = 1.23, (95% CI = 1.11, 1.35); RD = 0.14	RR = 1.25, (95% CI = 1.13, 1.39); RD = 0.15
HEC/MEC	5 mg	/	RD = 0.13	RD = 0.16	RR = 1.26, (95% CI = 1.06, 1.49); RD = 0.23	RR = 1.26, (95% CI = 1.06, 1.49); RD = 0.23	RR = 1.33, (95% CI = 1.09, 1.61); RD = 0.23

RD: risk difference; CC: complete control.

**Table 2:** The optimal dosing time for olanzapine.

Schema Group	Delayed Period	Acute Period	Overall Period
Olanzapine	CR = 79%,	CR = 95%,	CR = 78%,
	(95% CI = 75–83);	(95% CI = 93–97);	(95% CI = 74–82);
	CC = 78%,	CC = 94%,	CC = 76%,
	(95% CI = 74–82)	(95% CI = 92–97)	(95% CI = 72–81)
Placebo	CR = 66%,	CR = 89%,	CR = 64%,
	(95% CI = 66–71);	(95% CI = 85–92);	(95% CI = 59–69);
	CC = 64%,	CC = 88%,	CC = 61%,
	(95% CI = 59–67)	(95% CI = 85–91)	(95% CI = 56–66)
